# Characterization of a Pentacyclic Triterpene Acetyltransferase Involved in the Biosynthesis of Taraxasterol and ψ-Taraxasterol Acetates in Lettuce

**DOI:** 10.3389/fpls.2021.788356

**Published:** 2022-01-03

**Authors:** Han Suk Choi, Jung Yeon Han, Eun Ju Cheong, Yong Eui Choi

**Affiliations:** Department of Forest Resources, College of Forest and Environmental Sciences, Kangwon National University, Chuncheon, South Korea

**Keywords:** triterpene acetate, triterpene, triterpene acetyltransferase, taraxasterol, lettuce

## Abstract

Triterpenoids exist in a free state and/or in conjugated states, such as triterpene glycosides (saponins) or triterpene esters. There is no information on the enzyme participating in the production of triterpene esters from free triterpenes. Lettuce (*Lactuca sativa*) contains various pentacyclic triterpene acetates (taraxasterol acetates, ψ-taraxasterol acetates, taraxerol acetates, lupeol acetates, α-amyrin acetates, β-amyrin acetates, and germanicol acetate). In this study, we report a novel triterpene acetyltransferase (LsTAT1) in lettuce involved in the biosynthesis of pentacyclic triterpene acetates from free triterpenes. The deduced amino acid sequences of LsTAT1 showed a phylogenetic relationship (43% identity) with those of sterol *O*-acyltransferase (AtSAT1) of *Arabidopsis thaliana* and had catalytic amino acid residues (Asn and His) that are typically conserved in membrane-bound *O*-acyltransferase (MBOAT) family proteins. An analysis of LsTAT1 enzyme activity in a cell-free system revealed that the enzyme exhibited activity for the acetylation of taraxasterol, ψ-taraxasterol, β-amyrin, α-amyrin, lupeol, and taraxerol using acetyl-CoA as an acyl donor but no activity for triterpene acylation using a fatty acyl donor. Lettuce oxidosqualene cyclase (LsOSC1) is a triterpene synthase that produces ψ-taraxasterol, taraxasterol, β-amyrin and α-amyrin. The ectopic expression of both the *LsOSC1* and *LsTAT1* genes in yeast and tobacco could produce taraxasterol acetate, ψ-taraxasterol acetate, β-amyrin acetate, and α-amyrin acetate. However, expression of the *LsTAT1* gene in tobacco was unable to induce the conversion of intrinsic sterols (campesterol, stigmasterol, and β-sitosterol) to sterol acetates. The results demonstrate that the LsTAT1 enzyme is a new class of acetyltransferase belong to the MBOAT family that have a particular role in the acetylation of pentacyclic triterpenes and are thus functionally different from sterol acyltransferase conjugating fatty acyl esters.

## Introduction

Triterpenoids are diverse and abundant natural products that consist of six isoprene units, are categorized according to the structures of their triterpene rings and exhibit various biological and pharmacological activities (González-Coloma et al., 2011; Yang et al., 2020). Triterpenoids exist in a free state and in conjugated states, such as triterpene glycosides (saponins) or triterpene esters (Thimmappa et al., 2014). Triterpene glycosylation or acylation is an important phenomenon for diversifying the structure of triterpenes and may affect biological function (Osbourn et al., 2011). Acylated triterpenes esterified by acetic acid and fatty acids are very common in many plant species (Warnaar, 1987; Catharina et al., 2013; Hill and Connolly, 2017; Choi et al., 2020). Triterpene acetates showed higher antifungal and antibacterial activities than free triterpenes (do Nascimento et al., 2014; Javed et al., 2021). Triterpene acetates are found in the soil of plant vegetation and can be used as potential chemotaxonomic markers of Asteraceae to track past vegetation changes (Lavrieux et al., 2011; Nakamura, 2019).

Despite the widespread occurrence of triterpene esters in plants, there is little or no information on the genes participating in the production of triterpene esters from free triterpenes. Only some enzymes responsible for catalyzing the synthesis of sterol esters were identified. Sterols in plants (phytosterols) belong to the family of triterpenes and are composed of a tetracyclic ring and a side chain linked at position C-17 (Moreau et al., 2018). Both triterpenoids and phytosterols originate from a common precursor, 2,3-oxidosqualene (Phillips et al., 2006). Phytosterols also appear as either free sterols or steryl esters (Ferrer et al., 2017). Esterification of sterols is believed to play a role in storage pool of sterols (Korber et al., 2017). Biochemical studies have suggested that phospholipids and/or neutral lipids could serve as acyl donors in sterol ester synthesis (Duperon et al., 1984; Nyström et al., 2012). Sterol acyltransferases in plants can be categorized into two main groups, namely, acyl-CoA:sterol acyltransferases (ASAT; EC 2.3.1.26) and phospholipid:sterol acyltransferases (PSAT; EC 2.3.1.43) (Banas et al., 2005; Korber et al., 2017). An Arabidopsis ASAT enzyme (AtASAT1) acylates sterols using saturated fatty acyl-CoAs as acyl donors and possesses a certain degree of specificity for a sterol substrate, such as cycloartenol (Chen et al., 2007). Recently, Huang et al. (2019) reported that the AtASAT1 (THAA3) enzyme can convert two tricyclic triterpenes (thalianol and arabidiol) into triterpene fatty acid esters. AtASAT1 belongs to the superfamily of membrane-bound *O*-acyltransferases (MBOATs) composed of gene members encoding a variety of acyltransferase enzymes (Chen et al., 2007). The MBOAT protein family contains multiple transmembrane domains and share two active site residues, histidine and asparagine (Hofmann, 2000). Arabidopsis phospholipid:sterol acyltransferase (AtPSAT1, At1g04010) acylates various sterols (cholesterol, campesterol, sitosterol, and stigmasterol) and sterol intermediates using phosphatidylethanolamine as an acyl donor but does not acylate triterpenes such as lupeol and β-amyrin (Banas et al., 2005). Interestingly, homogenates of rabbit and human liver contain acyl-CoA:triterpene acyltransferase (ATAT) activity, which esterifies triterpenes absorbed from the diet by fatty acids, and has been demonstrated only at an enzymatic level (Tabas et al., 1991).

Serine carboxypeptidase-like (SCPL) proteins have recently been reported as plant acyltransferases. The oat SCPL protein SCPL1 (Sad7) encodes an SCPL acyltransferase needed for the acylation of avenacin (triterpene saponin) (Mugford et al., 2009). The SCPL1 enzyme is able to catalyze the acylation of avenacin into *N*-methyl anthraniloyl- and benzoyl-derivatized forms. Plant BAHD acyltransferase is a family of acyl CoA-utilizing enzymes involved in the production of volatile esters, anthocyanins, and phytoalexins (D’Auria, 2006). Two BAHD acyltransferases, thalianol acyltransferase (THAA) 1 and THAA2, catalyze the acetylation of the thalianol backbone (tricyclic triterpene) at the C-15 and C-3 hydroxy groups, respectively (Huang et al., 2019). Moreover, THAA2 can accept a broad range of triterpene substrates and specifically targets the C-3 hydroxy group of triterpenes (α-amyrin, β-amyrin, lupeol, and arabidiol) for acetylation (Bai et al., 2021).

In the present work, we isolated a triterpene acetyltransferase gene (*LsTAT1*) from the lettuce (*L. sativa*) transcriptome database by screening gene expression after elicitor treatment [methyl jasmonate (MeJA)]. The lettuce LsTAT1 enzyme is functionally characterized as a pentacyclic triterpene acetyltransferase, particularly for the production of taraxasterol acetates and ψ-taraxasterol acetates, based on an analysis of its enzyme activity, its heterologous expression in yeast, and its expression in tobacco plants.

## Results

### Analysis of Triterpenes and Triterpene Acetates in Different Lettuce Cultivars

**FIGURE 1:**
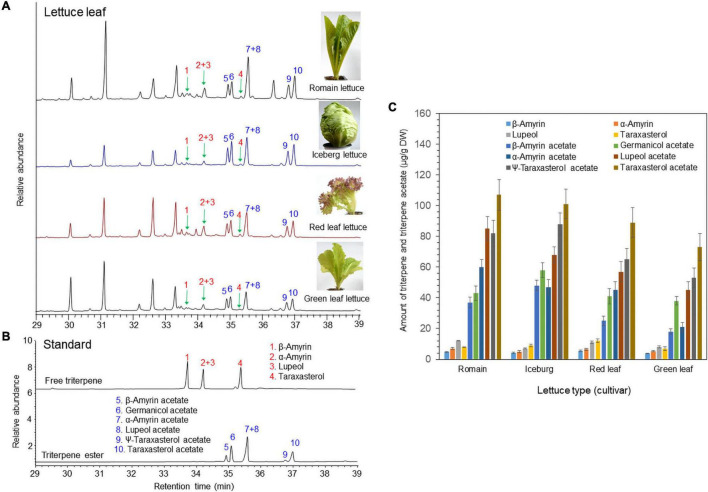
Identification of free triterpenes and triterpene esters in the leaves of four lettuce cultivars by gas chromatography–mass spectrometry (GC/MS) analysis. **(A)** Total ion chromatogram (TIC) of leaf extracts from four different lettuce cultivars. **(B)** TIC of authentic triterpene and triterpene ester standards. **(C)** Analysis of triterpenes and triterpene acetates in leaves of four lettuce cultivars.

**FIGURE 2:**
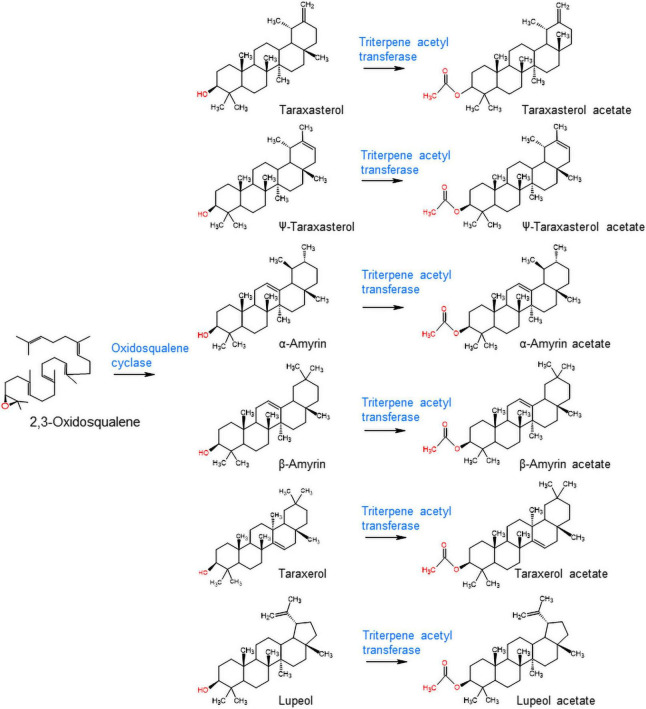
Biosynthesis of triterpene acetates from free triterpenes by oxidosqualene cyclase and triterpene acetyltransferase in lettuce.

The triterpene profiles in the leaves of four different lettuce cultivars (romaine lettuce, iceberg lettuce, red leaf lettuce, and green leaf lettuce) were determined by gas chromatography–mass spectrometry (GC/MS). Among the 12 triterpenes, six compounds were detected as triterpene esters (α-amyrin acetate, β-amyrin acetate, lupeol acetate, ψ-taraxasterol acetate, taraxasterol acetate, and germanicol acetate) as major constituents in the leaves of all four cultivars (Figures 1A,B). The other four compounds were found to be free triterpenes (β-amyrin, α-amyrin, lupeol, and taraxasterol). All six triterpene esters were esterified by acetic acid, as identified by a comparison with standard triterpene acetate compounds (Figure 1). The peak heights and areas of the triterpene acetates were generally higher than those of the free triterpenes (Figures 1A,B), which indicated that triterpene acetates are more highly accumulated than free triterpenes (Figure 1C). The occurrence of various triterpene acetates in lettuce may be due to the presence of triterpene acetyltransferases that convert free triterpenes into triterpene acetates (Figure 2).

### Isolation and Expression of Triterpene Acetyltransferase in Lettuce

**FIGURE 3:**
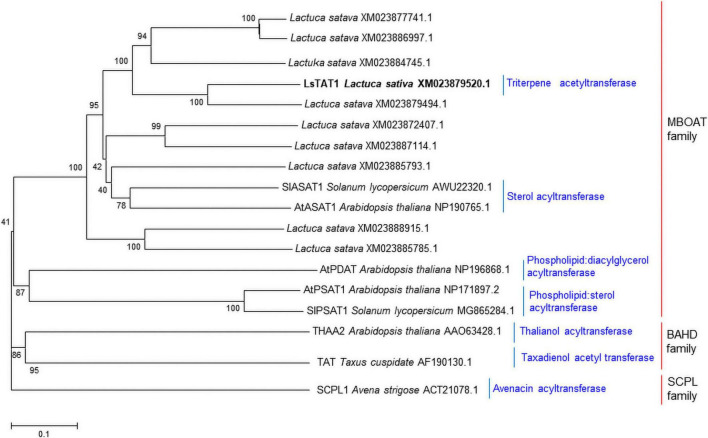
Phylogenetic trees of the 10 putative sterol *O*-acyltransferases isolated from lettuce and other functionally characterized acyltransferases in plants. Phylogenetic trees of plant acyltransferase showing the distances between each clone and group were constructed using the program ClustalW. The distance between each clone was calculated by the neighbor-joining method with a bootstrap of 1,000 replications using Mega 5 software. The number at each node is the bootstrap value in percentage.

We hypothesized that triterpene acetyltransferases in lettuce may be evolutionarily related to sterol *O*-acyltransferase in plants because sterol and triterpenes are derived from a common biosynthetic precursor (2,3-oxidosqualene). Interestingly, many acyl-CoA:sterol acyltransferase-like sequences in lettuce were registered in the NCBI GenBank database. Initially, the 10 sequences with similarity to acyl-CoA:sterol acyltransferases (*AtASAT1* and *SlASAT1*) were retrieved from the lettuce (crisphead-type lettuce cultivar, Salinas) NCBI database (BioProject: PRJNA432228). A phylogenetic analysis of the deduced amino acid sequences revealed that three sequences (XM_023872407.1, XM_023887114.1, and XM_023885793.1) were grouped into acyl-CoA:sterol acyltransferase (*AtASAT1* and *SlASAT1*) (Figure 3), but the other eight sequences were positioned in subgroups independent of the sequences of *AtASAT1* and *SlASAT1* (Figure 3).

**FIGURE 4:**
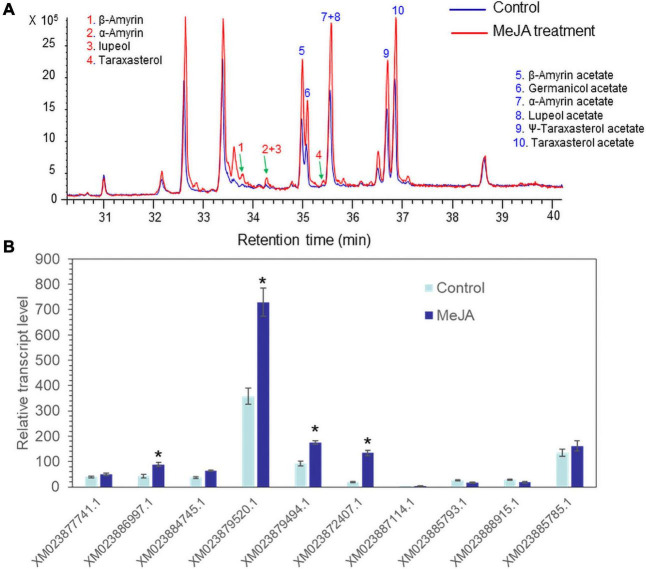
Accumulation of triterpenes and triterpene acetates and *LsTAT1* mRNA in lettuce seedlings treated with and without 100 μM methyl jasmonate (MeJA) for 3 days. **(A)** GC chromatograms of extracts from control seedlings (blue line) and MeJA-treated seedlings (red line). **(B**) Qantitative polymerase chain reaction (qPCR) analysis of the expression of 10 selected genes in lettuce seedlings after treatment with 100 μM MeJA for 3 days. The data were normalized to the β-actin gene expression level. The vertical bars indicate the SEs based on three biological replicates. The asterisks indicate significant differences from the control (Student’s *t*-test, *p* < 0.01).

Methyl jasmonate acts as an effective elicitor of secondary metabolite production in plants (Cheong and Choi, 2003). MeJA treatment can trigger the biosynthesis of a variety of defense chemicals including triterpenes and saponins (Lee et al., 2004; Misra et al., 2014; Singh and Dwivedi, 2018). Seedlings of lettuce were treated with 100 μM MeJA. GC/MS analysis revealed that all triterpene acetates (α-amyrin acetate, β-amyrin acetate, lupeol acetate, ψ-taraxasterol acetate, taraxasterol acetate, and germanicol acetate) were enhanced by treatment with 100 μM MeJA for 3 days (Figure 4A). A qantitative polymerase chain reaction (qPCR) analysis of 10 isolated lettuce sequences in the control and MeJA-treated lettuce seedlings revealed that one sequence (XM023879520.1) was most highly expressed among the other eight genes and strongly responded to MeJA treatment (Figure 4B). Thus, we selected XM023879520.1 as the best candidate sequence to investigate the functional properties of its enzyme.

Using primer pairs for the XM_023879520.1 sequence, a new open reading frame (ORF) sequence was obtained by sequencing the polymerase chain reaction (PCR) products from cDNAs of a green leaf-type lettuce cultivar (*L. sativa* var. crispa cv. Cheongchima). The ORF region of the new sequence showed a 2-bp difference from the original XM_023879520.1 sequence from Salinas lettuce, which is registered as the *LsTAT1* in GenBank (MZ268019). The full-length cDNA clone of *LsTAT1* is 1,062 bp, encodes a protein with 353 amino acids, and has a predicted molecular mass of 40.85 kDa. The deduced amino acid sequence of LsTAT1 was found to exhibit 43% identity with that of AtASAT1 (Supplementary Figure 1).

### *In vitro* Enzymatic Activity of LsTAT1

**FIGURE 5:**
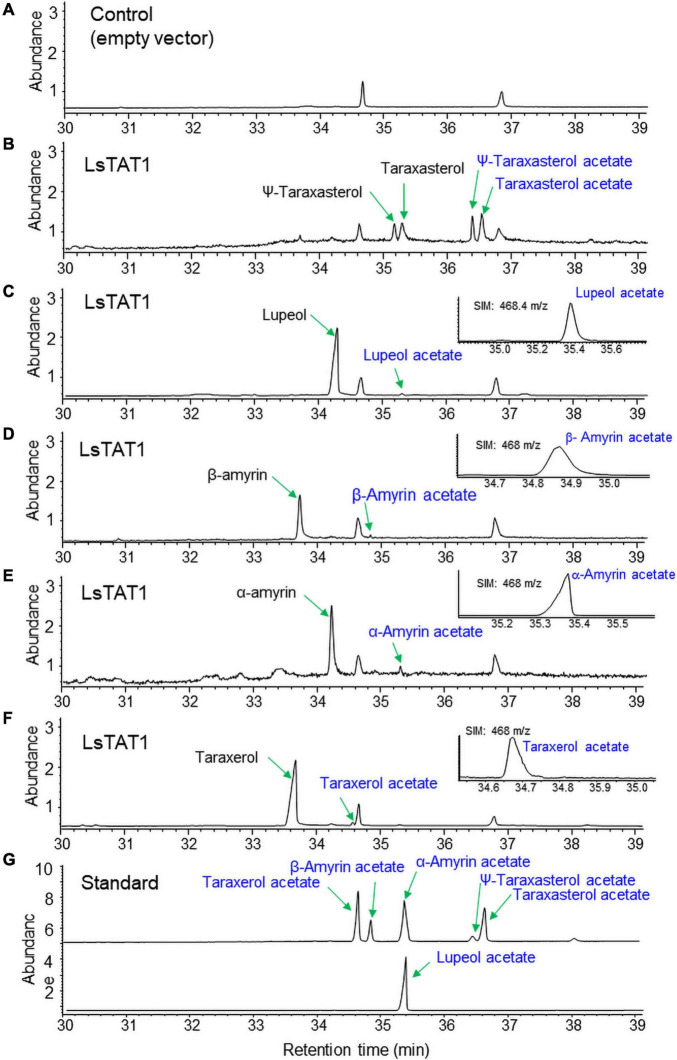
*In vitro* conversion of triterpenes to triterpene acetates by the reaction of microsomes from yeast expressing *Ls*TAT1. **(A)** Gas chromatography–mass spectrometry (GC/MS) chromatograms of the control of an assay with the microsome of yeast cells with an empty vector. **(B–F)** GC chromatograms of the reaction products of microsomes with taraxasterol and ψ-taraxasterol **(B)**, lupeol **(C)**, β-amyrin **(D)**, α-amyrin **(E)**, and taraxerol **(F)**. **(G)** GC chromatograms of standards of triterpene acetates. The inserted panels **(C–F)** are single ion mode (SIM) chromatograms of lupeol acetate, taraxerol acetate, β-amyrin acetate, and α-amyrin acetate.

**FIGURE 6:**
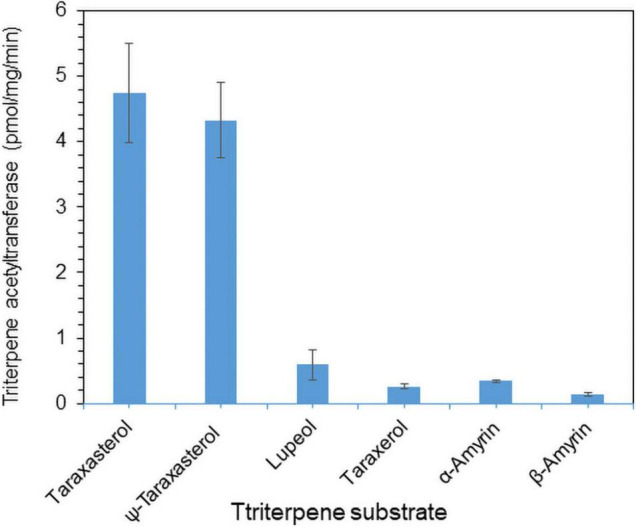
*In vitro* conversion rate of triterpenes into triterpene acetates catalyzed by the LsTAT1 enzyme. The enzyme activities are expressed as relative values of the conversion of triterpenes into triterpene acetates, and these values were analyzed by Gas chromatography–mass spectrometry (GC/MS) with selective ion monitoring. The values represent the means (*n* = 5) ± SEs.

To examine the enzyme activity of LsTAT1 in triterpene acetate production, microsomal fractions from a yeast strain (INVSc1) expressing the *LsTAT1* gene were incubated with various triterpenes using acetyl-CoA as an acyl donor. GC/MS analysis of a control consisting of an assay with the microsomal fractions of yeast cells with an empty vector showed two peaks that were derived from intrinsic sterol compounds of yeast microsomal protein extracts (Figure 5A). GC/MS analysis of extracts from triterpene and enzyme mixtures clearly revealed that the LsTAT1 enzyme is able to convert taraxasterol, ψ-taraxasterol, lupeol, taraxerol, β-amyrin, and α-amyrin into taraxasterol acetate, ψ-taraxasterol acetate, lupeol acetate, taraxerol acetate, β-amyrin acetate, and α-amyrin acetate, respectively (Figures 5B–F). The production of lupeol acetate, taraxerol acetate, β-amyrin acetate, and α-amyrin acetate with tiny peaks was confirmed with the single ion mode (SIM) (Figures 5C–F) and by comparing the mass fraction of the compounds with those of authentic standards (Supplementary Figure 3). All the produced triterpene acetates in the enzymatic reactions were matched with standard triterpene acetates (Figure 5G), all of which have a single acetyl group at the C-3 portion of triterpenes, and this finding indicated that the LsTAT1 enzyme can catalyze the acetylation of the C−3 hydroxyl group in the triterpene backbone. The enzyme exhibited higher activity for taraxasterol- and ψ-taraxasterol acetate formation than other triterpene acetates, and more than 40% of taraxasterol- and ψ-taraxasterol triterpenes were converted into taraxasterol acetate and ψ-taraxasterol acetate, respectively (Figure 6). However, the conversion rate of other triterpenes (lupeol, taraxerol, β-amyrin, and α-amyrin) into their acetate forms did not exceed 10% (Figure 6). To examine the acylation activity of LsTAT1 using fatty acyl-CoA, the enzyme was incubated with palmitoyl-CoA (16:0) and stearoyl-CoA (18:0) as acyl donors and lupeol as an acyl accepter. However, LsTAT1 did not show triterpene acylation activity using fatty acyl-CoA (Supplementary Figure 4).

### Co-expression of Lettuce Oxidosqualene Cyclase (*LsOSC1*) and *LsTAT1* in Yeast

**FIGURE 7:**
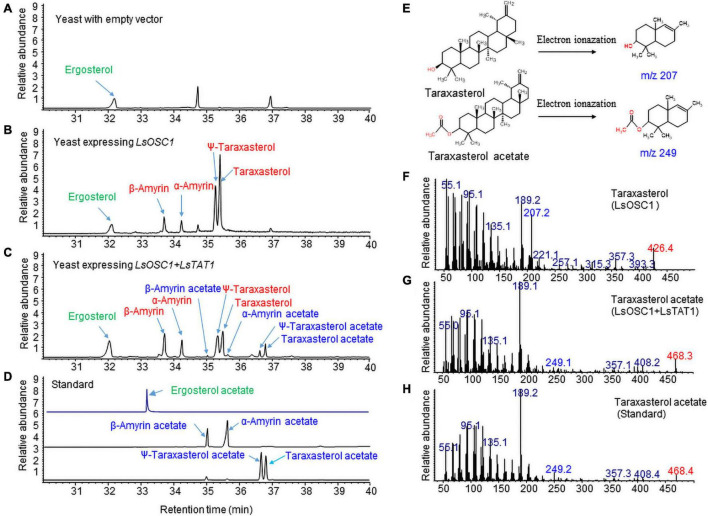
Gas chromatography (GC) analysis of triterpene acetates extracted from yeast co-expressing both the *LsOSC1* and *LsTAT1* genes. **(A)** Chromatogram of control yeast transformed with the empty vector. **(B)** Chromatogram of triterpene products (β-amyrin, α-amyrin, ψ-taraxasterol, taraxasterol, and dammarenediol-II) in yeast transformed with *LsOSC1*. **(C)** Chromatogram of two triterpene acetates (taraxasterol acetate and ψ-taraxasterol acetate) in yeast transformed with both the *LsOSC1* and *LsTAT1* genes. **(D)** GC chromatograph of authentic standards of β-amyrin acetate, α-amyrin acetate, ψ-taraxasterol acetate, and taraxasterol acetate. **(E)** Structures of taraxasterol and taraxasterol acetate and their mass fragment ions by Electron Ionization-mass spectrometry (EI-MS). **(F)** MS spectrum of taraxasterol produced in yeast expressing *LsOSC1*. **(G)** MS spectrum of taraxasterol acetate produced in yeast expressing *LsOSC1* and *LsTAT1*. **(H)** MS spectrum of the taraxasterol acetate standard.

We previously identified an LsOSC1 enzyme (GenBank accession number, MN107542) in lettuce as a multifunctional oxidosqualene cyclase that produces multiple triterpenes (mainly taraxasterol and ψ-taraxasterol together with minor amounts of β-amyrin, α-amyrin, and dammarenediol-II) (Choi et al., 2020). The *LsOSC1* and *LsTAT1* genes were cloned into the pESC-URA yeast expression vector with double gene expression cassettes. The total ion chromatogram (TIC) obtained by GC/MS revealed that the expression of *LsOSC1* alone in yeast produced four new triterpene peaks at retention times between 32 and 38 min, which were the same retention times as those of the β-amyrin, α-amyrin, ψ-taraxasterol, and taraxasterol standards (Figure 7B). The co-expression of *LsOSC1* and *LsTAT1* in yeast revealed the production of four additional novel triterpene acetate peaks at retention times between 32 and 38 min, which had the same retention times as ψ-taraxasterol acetate, taraxasterol acetate, β-amyrin acetate, and α-amyrin acetate (Figure 7C). In the control yeast with only an empty vector, no triterpene peak with the exception of yeast products such as ergosterols was detected (Figure 7A). Electron Ionization-mass spectrometry (EI-MS) showed *m/z* 207 [M-H]^+^ and *m/z* 249 [M-H]^+^ ion fragments characteristic of taraxasterol and taraxasterol acetate, respectively (Figures 7E–G; Choi et al., 2020). The mass spectrum of taraxasterol acetate (Figure 7G) produced in yeast expressing *LsOSC1* and *LsTAT1* matched that of standard taraxasterol acetate (Figure 7H). To investigate the possible activity of the LsTAT1 enzyme on the production of ergosterol acetate, the TIC obtained in the SIM for the molecular ion (*m/z* 438.7) of ergosterol acetate was analyzed together with the TIC of the standard compound (ergosterol acetate). However, ergosterol acetate was not detected in yeast expressing *LsOSC1* and *LsTAT1* (Figure 7D).

### Triterpene Acetate Production in Transgenic Tobacco Overexpressing *LsOSC1* and *LsTAT1*

Transgenic tobacco plants co-overexpressing *LsOSC1* and *LsTAT1* under the control of the CaMV35 promoter were constructed. The transgenic tobacco lines were confirmed by genomic PCR of the *BAR* (phosphinothricin *N*-acetyltransferase), *LsOSC1*, and *LsTAT1* genes (Supplementary Figure 5). No signal was detected with wild-type tobacco (Supplementary Figure 5).

**FIGURE 8:**
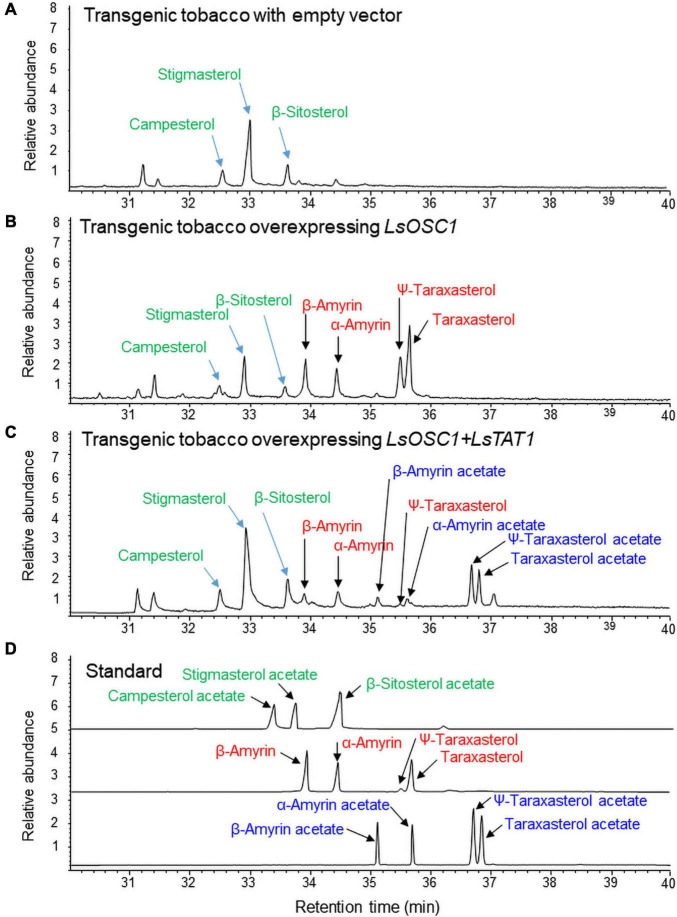
Gas chromatography (GC) analysis of triterpene acetates extracted from leaves of transgenic tobacco co-expressing both the *LsOSC1* and *LsTAT1* genes. **(A)** Chromatogram of leaf extracts of control tobacco transformed with the empty vector. **(B)** Chromatogram of triterpene products (β-amyrin, α-amyrin, ψ-taraxasterol, taraxasterol, and dammarenediol-II) in transgenic tobacco overexpressing the *LsOSC1* gene. **(C)** Chromatogram of triterpene acetates (taraxasterol acetate and ψ-taraxasterol acetate) in transgenic tobacco overexpressing both the *LsOSC1* and *LsTAT1* genes. **(D)** GC chromatograph of authentic standards of β-amyrin acetate, α-amyrin acetate, ψ-taraxasterol acetate, and taraxasterol acetate.

The triterpene content in the leaf extracts of transgenic tobacco was analyzed by GC/MS. Transgenic tobacco overexpressing both *LsOSC1* and *LsTAT1* produced four free triterpenes (α-amyrin, β-amyrin, ψ-taraxasterol, and taraxasterol) and four triterpene esters (α-amyrin acetate, β-amyrin acetate, ψ-taraxasterol acetate, and taraxasterol acetate) (Figure 8C). Transgenic tobacco overexpressing *LsOSC1* alone produced four triterpene peaks at retention times between 32 and 38 min (Figure 8B), which were the same retention times as those of the β-amyrin, α-amyrin, ψ-taraxasterol, and taraxasterol standards (Figure 8D). With wild-type tobacco, only phytosterols (campesterol, stigmasterol, and β-sitosterol) were detected (Figure 8A). To review the possible esterification of endogenous free phytosterols (campesterol, stigmasterol, and β-sitosterol) into sterol acetates by the LsTAT1 enzyme, we analyzed the presence of peaks of campesterol acetate, stigmasterol acetate, and β-sitosterol acetate in the GC chromatograms through comparisons with chromatograms of authentic standards of sterol esters. However, no peaks for campesterol acetate, stigmasterol acetate, and β-sitosterol acetate were detected (Figure 8C).

## Discussion

The types of conjugated triterpenes show variations among different plant taxa. In Compositae (Asteraceae), triterpenes mainly exist as acetated triterpenes compared to free triterpenes (Huber et al., 2015; Bahadir-Acikara et al., 2018; Choi et al., 2020). GC/MS analysis of leaf extracts of four lettuce cultivars revealed the accumulation of various types of pentacyclic triterpene acetates in leaves and these triterpene acetates as major constituents. Triterpene acetates showed higher antifungal and antibacterial activities than free triterpenes (do Nascimento et al., 2014; Javed et al., 2021). Thus, triterpene acetylation from free-triterpenes in plants may be an important biological defense mechanism against microorganisms.

Pentacyclic triterpenes and sterol (cycloartenol) in plants show highly similar triterpene skeletal structures and are derived from the common pathway of terpenoid biosynthesis up to 2,3-oxidosqualene. *Arabidopsis* sterol acyltransferase (AtASAT1, At3g51970, and THAA3) exhibits a preference for fatty acyl-CoAs as acyl donors and cycloartenol, thalianol, and arabidiol as acyl acceptor molecules (Chen et al., 2007; Huang et al., 2019). Interestingly, many ASAT-like mRNA sequences have been deposited in the lettuce transcriptome in the NCBI database. Among the 10 selected mRNA ASAT-like sequences in the lettuce transcriptome, one gene sequence (XM_023879520.1) was selected as a candidate gene because the expression of the gene was highly enhanced by treatment with MeJA, which is a strong elicitor of triterpene and saponin biosynthesis (Singh and Dwivedi, 2018).

A phylogenetic analysis revealed that the LsTAT1 enzyme was positioned in different subgroups of AtASAT1. The deduced amino sequence of the LsTAT1 enzyme showed only 43% identity with that of AtASAT1. LsTAT1 has catalytic amino acid residues (Asn and His) that are typically conserved in MBOAT family proteins (Supplementary Figure 1). MBOAT enzymes contain multiple transmembrane domains (Hofmann, 2000). The visualization of LsTAT1 protein topology using Protter^1^ (Omasits et al., 2014) revealed that LsTAT1 is a membrane-bound protein with five transmembrane helices in the protein sequence (Supplementary Figure 2B). These results suggest that LsTAT1 enzyme belongs to the MBOAT family of proteins because they have multiple transmembrane domains and catalytic amino acid residues (Asn and His).

Sterol esters are synthesized in the endoplasmic reticulum and found in lipid bodies in the cytoplasm (Korber et al., 2017). Esterification of sterols is believed to play a role in storage pool of sterols. AtSAT1 protein is localized in the endoplasm reticulum (Lara et al., 2018). In contrast to AtSAT1, tomato (*Solanum lycopersicum*) SAT1 (SlASAT1) protein resides in the plasma membrane (Lara et al., 2018). The subcellular localization of LsTAT1 was predicted using DeepLoc-1.0 software (Almagro Armenteros et al., 2017), and LsTAT1 was localized in the endoplasmic reticulum (Supplementary Figure 2A). This result indicates that pentacyclic triterpene acetates in lettuce may be synthesized in the endoplasmic reticulum and then accumulated in lipid bodies. Unlike to sterol esters maintaining free sterol homeostasis in the cell membranes, pentacyclic triterpene esters are considered to be act as defensive compounds against microbes and pests (Osbourn et al., 2011). Pentacyclic triterpene acetylation from free triterpenes may confer different antimicrobial properties.

Functional characterization of the LsTAT1 enzyme revealed that LsTAT1 exhibited no activity for triterpene acylation using long-chain fatty acyl-CoA. This result indicates that LsTAT1 is functionally different enzyme from sterol acyltransferases such as AtASAT1. LsTAT1 is a pentacyclic triterpene acetyltransferase with particularly high chemical affinity to taraxasterol and ψ-taraxasterol for acetylation.

An *Arabidopsis* acetyltransferase (THAA2) enzyme belonging to the BAHD acyltransferase superfamily has thalianol (tricyclic triterpene) acetyltransferase activity (Huang et al., 2019) and can accept various other triterpene substrates (α-amyrin, β-amyrin, lupeol, and arabidiol) for acetylation (Bai et al., 2021). We screened the genes homologous to the *Arabidopsis* THAA2 enzyme in lettuce transcriptome sequences in NCBI but failed to find suitable genes for further analysis.

The *LsOSC1* gene is a multifunctional triterpene synthase that produces five triterpenes, namely, taraxasterol, Ψ-taraxasterol, α-amyrin, β-amyrin, and dammarenediol-II (Choi et al., 2020). The role of the LsTAT1 enzyme in the production of triterpene acetates was also demonstrated by the heterologous expression of both the *LsTAT1* and *LsOSC1* genes in yeast. The co-expression of *LsOSC1* and *LsTAT1* in yeast can convert all four free triterpenes (taraxasterol, Ψ-taraxasterol, α-amyrin, and β-amyrin) to triterpene acetates. Transgenic yeasts co-expressing *LsOSC1* and *LsTAT1* exhibited a conspicuous ergosterol (yeast sterol triterpene) peak in the GC/MS chromatogram. However, no signal for ergosterol acetate was detected in either the TIC or SIM chromatograms obtained from extracts of transgenic yeasts. This result indicates that *LsTAT1* may not have activity for yeast sterol acetylation.

The two lettuce genes *LsOSC1* and *LsTAT1* were overexpressed in transgenic tobacco to investigate the function of the LsTAT1 enzyme in plants. In transgenic tobacco, most taraxasterol and Ψ-taraxasterol were converted into taraxasterol acetate and Ψ-taraxasterol acetate, respectively. However, the remaining α-amyrin and β-amyrin were not fully converted into α-amyrin acetate and β-amyrin acetate, respectively. This result suggests that the LsTAT1 enzyme is mainly responsible for the production of taraxasterol acetate and Ψ-taraxasterol acetate.

Some plants produce various triterpene esters esterified by fatty acids (Ukiya et al., 2001; Fingolo et al., 2013). In tobacco leaf extracts, prominent amounts of phytosterols (campesterol, stigmasterol, and β-sitosterol) were detected in the GC chromatograms. However, no signals for campesterol acetate, stigmasterol acetate, or β-sitosterol acetate were detected in the GC chromatograms of transgenic tobacco overexpressing *LsOSC1* and *LsTAT1*. This result implies that LsTAT1 has no phytosterol esterification activity.

## Conclusion

In conclusion, the lettuce LsTAT1 enzyme is a new triterpene acetyltransferase belonging to the MBOAT family, particularly for the biosynthesis of taraxasterol acetates, and is functionally different from sterol acyltransferases conjugating long fatty acyl-CoAs.

## Materials and Methods

### Isolation of *LsTAT1* Genes in Lettuce Transcriptome and Phylogenetic Analysis

Two functionally characterized acyl-CoA:sterol *O*-acyltransferases, *Arabidopsis* AtASAT1 (At3g51970, THAA3) and tomato SlASAT (MG865283.1), and several acyl-CoA-sterol *O*-acyltransferase-like genes in lettuce (Figure 3) were selected from the NCBI nucleotide sequence database of lettuce registered by Reyes-Chin-Wo et al. (2017). Initially, 10 putative acyl-CoA sterol *O*-acyltransferase-like genes in lettuce (crisphead-type lettuce cultivar, Salinas, CA, United States) were retrieved from the database. We also isolated one mRNA sequence (XM_0238479520.1) from the lettuce sequence database. Using primer pairs for the XM_023879520.1 sequence, the ORF sequence was obtained by sequencing the PCR products from cDNAs of a green leaf-type lettuce cultivar (*L. sativa* var. crispa cv. Cheongchima). The ORF region of the new *LsTST1* sequence showed a 2-bp difference from that of the original XM_023879520.1 sequence from Salinas lettuce, which is registered as the *LsTAT1* gene in GenBank (MZ268019).

Multiple sequence alignments were performed using the CLUSTAL W program (Thompson et al., 1997). The subcellular localization of the selected genes was further predicted using DeepLoc-1.0 (Almagro Armenteros et al., 2017). The predictions of the transmembrane helices and protein topology of the enzyme was analyzed with Protter (Omasits et al., 2014).

### Phylogenetic Analysis of Amino Acid Sequences

The deduced amino acid sequences of 10 ASAT-like genes of lettuce and related genes in other plants obtained from NCBI GenBank were subjected to a phylogenetic analysis.

The analysis was conducted using the neighbor-joining method with MEGA 6.0 software (Tamura et al., 2013). A bootstrap analysis with 1,000 replicates was performed to assess the strength of the nodes in the tree (Felsenstein, 1985).

### Elicitor (Methyl Jasmonate) Treatment and Qantitative Polymerase Chain Reaction Analysis in Lettuce Seedlings

Two-week-old lettuce seedlings were transferred to liquid medium with 0 and 100 μM MeJA for 3 days. Total mRNA from the lettuce seedlings was isolated and then converted to cDNA using an ImProm-II Reverse Transcription System (Promega, Madison, WI, United States). qPCR was performed using a real-time PCR system with a SYBR Green PCR kit (Qiagen, Germany). The primers for 10 isolated sequences and β-actin are shown in Supplementary Table 1. The expression of target genes was calculated using the ^–ΔΔ^ *C*_*T*_ method (Livak and Schmittgen, 2001). The lettuce β-actin gene was used for normalization. The data are presented as the means ± SEs from at least three independent experiments.

### Ectopic Expression of *LsTAT1* in Yeast

Total RNA from the leaves of lettuce (*L. sativa* var. crispa cv. Cheongchima) was extracted using an RNeasy plant mini kit (Qiagen, Germany) following the manufacturer’s instructions. cDNA was obtained from the extracted mRNA using an ImProm-2 Reverse Transcription System (Promega Co., Madison, WI, United States). To construct an expression plasmid vector for yeast, the ORF from *LsTAT1* amplified from cDNA was cloned into the pYES2.1/V5-His-TOPO vector (Invitrogen, Thermo Fisher Scientific, Waltham, MA, United States) and transformed into *Escherichia coli*. The primer pairs used to isolate the *LsTAT1* cDNAs are shown in Supplementary Table 1. The ORF was then ligated to the GAL1 promoter in the sense orientation. After confirmation of the nucleotide sequence of the inserted DNA, *LsTAT1* and an empty vector were expressed in the *Saccharomyces cerevisiae* strain INVSc1 (Invitrogen). INVSc1 yeast cells were transformed using a modified lithium acetate procedure, as described previously (Gietz et al., 1992). Transformed cells were selected by SC-T (SC minimal medium lacking tryptophan) and subcultured on YPG medium (Kribii et al., 1997).

### Membrane Protein Extraction and Enzyme Assay

Microsomal proteins were extracted from yeast after galactose induction for 1 day. The yeast culture was centrifuged (2,000 × *g*, 10 min), and the pellet was resuspended in 50 ml of TEK (100 mM KCl in 50 mM Tris–HCl with 1 mM EDTA) and centrifuged at 6,100 × *g* and 4°C for 5 min. The yeast pellets were ground by agitation with glass beads in 5 mM EDTA, 5 mM Tris–HCl, and 0.6 M sorbitol, pH 7.5. The resultant homogenate was spun at 10,000 × *g* for 15 min, and the supernatant was transferred to a new tube and centrifuged by ultracentrifugation at 100,000 × *g* and 4°C for 60 min. The pellet was resuspended in 0.1 M potassium phosphate buffer (pH 7.4), 20% glycerol, and 20 mM β-mercaptoethanol for enzyme assay. The protein concentration was measured using the Bradford protein assay.

The enzymatic activity of LsTAT1 was tested in a total volume of 400 μl of 0.1 M potassium phosphate buffer (pH 7.4), 1 mM dithiothreitol (DTT), 5 mM MgCl_2_, 200 μM substrates (α- and β-amyrin, taraxasterol, ψ-taraxasterol, taraxerol, and lupeol), 100 μM acyl donors (acetyl-CoA, palmitoyl-CoA, or stearoyl-CoA), and 200 μg of total microsomal proteins. A reaction without exogenous acetyl-CoA was employed as a control. The reaction mixture was incubated for 1 h at 30°C, and the reaction was extracted twice with the same volume of chloroform. The activity of LsTAT1 was determined by the formation of triterpene acetates from triterpenes per milligram of protein per minute. The reaction mixture was mixed with 100% chloroform by vortexing. After centrifugation, the chloroform layer was transferred to a new tube and filtered using a SepPak C-18 cartridge (Waters, Milford, MA, United States). The *in vitro* activity of the LsTAT1 enzyme on the conversion of triterpenes to triterpene acetates was monitored by GC/MS analysis.

### Co-expression of *LsTAT1* and *LsOSC1* in Yeast

The ORF of *LsOSC1* (GenBank accession number, MN107542.1) was amplified using PCR primers, as shown in Supplementary Table 1. The PCR products were cloned into a pYES2.1/V5-HIS-TOPO expression vector (Invitrogen, United States) under the control of the GAL1 promoter. *E. coli* were transformed with the recombinant vectors. Gene insertion into the vectors was confirmed by nucleotide sequencing of plasmids isolated from each colony. The isolated plasmid was inserted into yeast strain INVSc1 (MATa *his3*Δ*1 leu2 trp1-289 ura3-52*/MATα *his3*Δ*1 leu2 trp1-289 ura3-52*), which was obtained from Invitrogen (Carlsbad, CA, United States). The transformed yeasts were grown in 50 ml of synthetic complete medium without uracil (SC-U) containing 2% glucose. After 2 days, cells were collected and resuspended in SC-U medium with 2% galactose and then inducted at 30°C for 16 h. Thereafter, the cells were collected, refluxed with 80% MeOH and sonicated for 30 min at 40°C. After centrifugation, the supernatant was transferred to chloroform and vortexed. The chloroform layer was subsequently filtered using a SepPak C-18 cartridge (Waters Co., Milford, MA, United States) and analyzed by GC/MS.

The coding region of *LsTAT1* (GenBank accession No. GU183405) was amplified using the primer pairs mentioned above, subcloned into the pGEM-T Easy vector and sequenced. The ORF fragments were excised through *Not*I and inserted into the *Not*I site of the pYES3/CT vector by ligation to construct an expression vector (promoter: *GAL*1, selection marker: *TRP*1) (Invitrogen, Thermo Fisher Scientific, Waltham, MA, United States). The vector was introduced into yeast. Gene insertion into the vectors was confirmed by nucleotide sequencing of plasmids isolated from each colony. For cotransformation of the *LsTAT1* and *LsOSC1* genes in yeast, plasmids containing *LsTAT1* were transformed into INVSc1 yeast expressing *LsOSC1*, and the cells were cultured on medium without uracil and tryptophan. The culture conditions and methods for galactose induction and preparation of the triterpene fraction were the same as those described above. To analyse the production of triterpenes in transgenic yeasts, transgenic yeast cells harvested after centrifugation were mixed with 80% methanol and sonicated for 10 min. After centrifugation, the supernatant was mixed with 100% chloroform by vortexing. The chloroform layer was transferred to a new tube and filtered using a SepPak C-18 cartridge (Waters).

### *In planta* Expression of the *LsTAT1* and *LsOSC1* Genes

The ORF regions of *LsTAT1* (GenBank accession number, MZ268019) and *LsOSC1* (GenBank accession number, MN107542.1) were amplified using PCR primers, as shown in Supplementary Table 1. Both genes were cloned into the pCR8/GW/TOPO (Invitrogen, Carlsbad, CA, United States) vector and transferred into the destination vector pPZIP-Bar under the control of the double 35S CaMV promoter. Eventually, the overexpression construct harboring both the *LsTAT1* and *LsOSC1* genes was introduced into *Agrobacterium tumefaciens* GV3101 competent cells by the heat shock method. The construction of transgenic tobacco with the *LsTAT1* and *LsOSC1* genes was performed by *A. tumefaciens*-mediated transformation.

### Gas Chromatography–Mass Spectrometry Analysis

Seeds of four cultivars (romaine lettuce, iceberg lettuce, red leaf lettuce, and green leaf lettuce) of lettuce were purchased from Asia Seed Co. (Seoul, South Korea). At the two- to three-true-leaf stages, germinated plants were transplanted into plastic pots containing peat moss and perlite (3:1 v/v). The plants were grown in a greenhouse with a 16-h photoperiod and day and night temperatures of 24 and 18°C, respectively. When the plants had grown to the 10- to 12-leaf stage, the third leaves from the top were sampled for triterpene analysis.

To analyse the triterpene profiles in leaves of four lettuce cultivars, MeJA treated lettuce seedlings, and leaves of transgenic tobacco with the *LsTAT1* and *LsOSC1* genes, the leaves were air dried at 50°C in a drying oven. The milled powders (200 mg) from each of the samples were soaked in 100% methanol (1 ml) and sonicated for 30 min at a constant frequency of 20 kHz and a temperature of 40°C. The supernatant was collected by centrifugation and subsequently filtered using a SepPak C-18 cartridge (Waters Co., Milford, MA, United States).

For the GC/MS analysis prepared from the extracts mentioned above, an aliquot (5 μl) with a split injection (5:1) was obtained for analysis using a gas chromatograph (Agilent 7890A) equipped with an inert MSD system (Agilent 5975C) with a triple-axis detector and an HP-5 MS capillary column (30 m × 0.25 mm, film thickness of 0.25 mm). The inlet temperature was set to 250°C, and the column temperature was programmed to start at 150°C for 5 min, increase to 300°C at a rate of 5°C/min, and remain at 300°C for 20 min. The GC/MS conditions were set as follows: ionizing energy, 70 eV; ion source temperature, 230°C; and MS quad temperature, 150°C. The chromatogram peaks in the GC analysis were identified by comparison with a library database and authentic triterpene and triterpene acetate standards. Triterpenes and triterpene acetates were identified by comparing their retention times with those of authentic standards and mass fragmentation spectra.

### Ultra-Performance Liquid Chromatography Analysis

The *in vitro* activity of the *LsTAT1* enzyme on the conversion of lupeol to lupeol palmitate by the reaction of palmitoyl-CoA (16:0) as an acyl donor and lupeol as an acyl accepter was monitored by an ACQUITY ultra-performance liquid chromatograph (UPLC) (Waters Co., Milford, MA, United States) equipped with a Photodiode Array (PDA) detector.

A binary gradient method was used to vary the mobile phase composition over time. Mobile phase solvents A and B consisted of water and acetonitrile, respectively. Linear gradient elution at a flow rate of 0.5 ml/min was performed for analysis: initial 15% B from 0 to 0.5 min, linear gradient 15–45% B from 0.5 to 8 min, linear gradient 45–100% B from 8 to 10 min, and a final return to 15% B, which was maintained until 2 min. The total run time was 18 min, and the sample injection volume was 2 μl.

## Data Availability Statement

Sequence data from this article can be found in the GenBank databases under the following accession numbers: *LsTAT1 cDNA*, *MZ268019*; *LsOSC1 cDNA*, *MN107542.1*.

## Author Contributions

YC designed the research. HC and JH performed the research. EC analyzed the data. YC and HC wrote the manuscript. All authors contributed to the article and approved the submitted version.

## Conflict of Interest

The authors declare that the research was conducted in the absence of any commercial or financial relationships that could be construed as a potential conflict of interest.

## Publisher’s Note

All claims expressed in this article are solely those of the authors and do not necessarily represent those of their affiliated organizations, or those of the publisher, the editors and the reviewers. Any product that may be evaluated in this article, or claim that may be made by its manufacturer, is not guaranteed or endorsed by the publisher.

## Footnotes

1
https://wlab.ethz.ch/protter/start/
