# Supplementary material for: Characterization of a Pentacyclic Triterpene Acetyltransferase Involved in the Biosynthesis of Taraxasterol and ψ-Taraxasterol Acetates in Lettuce
Source: Front Plant Sci. 2022 Jan 3;12:788356. doi: 10.3389/fpls.2021.788356 (PMC8762322; doi:10.3389/fpls.2021.788356)
Supplement: Supplementary file 1 [file Data_Sheet_1.PDF]

```
LsTAT1          MEGEAYNFILDWSVAVASLFYCHSVGKFTAPGTTRFVALFPVMFLFFYLPLNLYTMFLCG    60
AtSAT1          ----MASFIKAWGLVIISLCYTFFIAKLVPKGIKRLILFFPVFLIFFIVPFLIYSLHLLG    56
                      .**  *.:.: ** * . :.*:.. * .*:: :***:::** :*: :*::.* *

LsTAT1          PTFFFISWLGSFKLVLYSFGKGPLSS-HPPLPLSHFISSACLPIKVKRNQEDTSDQITKR    119
AtSAT1          ITAFFIAWLANFKLLLFALGRGPLSSNHKPLSLPIFLAVSCLPIKIQLSPKPTKTHSHEG    116
                 * ***:**..***:*:::*:***** * **.*. *:: :*****:: . : *. :  :

LsTAT1          PQRSIIDYAPRVFLLLIAIKAYDYKANLHPLLLTSIYSYYIFFWLELILAVAASLARTLV    179
AtSAT1          STEGPLIYTIKAVFVVLIIKAYEYSTKLPEKVVLTLYAIHIYFALEIILAATAAAVRAMS    176
                . .. : *: :..:::: ****:*.::*   :: ::*: :*:* **:***.:*: .*::

LsTAT1          GVELEPQFDEPHQATSVQNFWGKRWNLMVSSILRPTVYHPSRAIFGRVVPERWVSVPAVF    239
AtSAT1          DLELEPQFNKPYLATSLQDFWGRRWNLMVTGILRPTVYEPSLQLFSVLGPN-YSQILAAF    235
                .:******::*: ***:*:***:******:.*******.**  :*. : *: : .: *.*

LsTAT1          TTFVVSGIMHEIIFYYLGRLTPTWEVTWFFVIQGVWVGMEIVIKKTIGRQFKPQRVVSRV    299
AtSAT1          GTFVVSGIMHELIFFYMGRLRPDWKMMWFFLINGFCTTVEIAIKKTINGRWRFPKAISQV    295
                 **********:**:*:*** * *:: ***:*:*. . :**.*****. :::  :.:*:*

LsTAT1          LTLVFVITTSFWLFFPPFMRLNPFARGCRELMAFAGLFKHGYLIRPDEYSCPYF          353
AtSAT1          LTLTFVMVTALWLFLPEFNRCNIVEKALDEYAAIGAFAVEVRRKLTAYLF----          345
                ***.**:.*::***:* * * * . :.  *  *:..:  .     .
```

Figure S1. Sequence alignment of LsTAT1 and AtSAT1 proteins. Amino acid sequences were aligned using the Clustal Omega multiple sequence alignment tool (http://www.ebi.ac.uk/Tools/msa/clustalo/). Amino acid residues are numbered on the right. Star markers denote residues conserved in the two sequences. Colons indicate conservation between amino acids of strongly similar properties whereas periods indicate conservation between amino acids of weakly similar properties. Hyphens indicate gaps introduced to optimize the alignment. The catalytic amino acid residues (Asn and His) are marked in blue. The RxWNxxVxxxLxxxVY motif in red rectangular line believed to be crucial for acyl-CoA binding is also found in LsTAT1. The sequences shown have the following GenBank accession numbers: MZ268019 (LsTAT1) and NM_115056.3 (AtSAT1).
