# Supplementary material for: Characterization of a Pentacyclic Triterpene Acetyltransferase Involved in the Biosynthesis of Taraxasterol and ψ-Taraxasterol Acetates in Lettuce
Source: Front Plant Sci. 2022 Jan 3;12:788356. doi: 10.3389/fpls.2021.788356 (PMC8762322; doi:10.3389/fpls.2021.788356)
Supplement: Supplementary file 2 [file Data_Sheet_2.PDF]

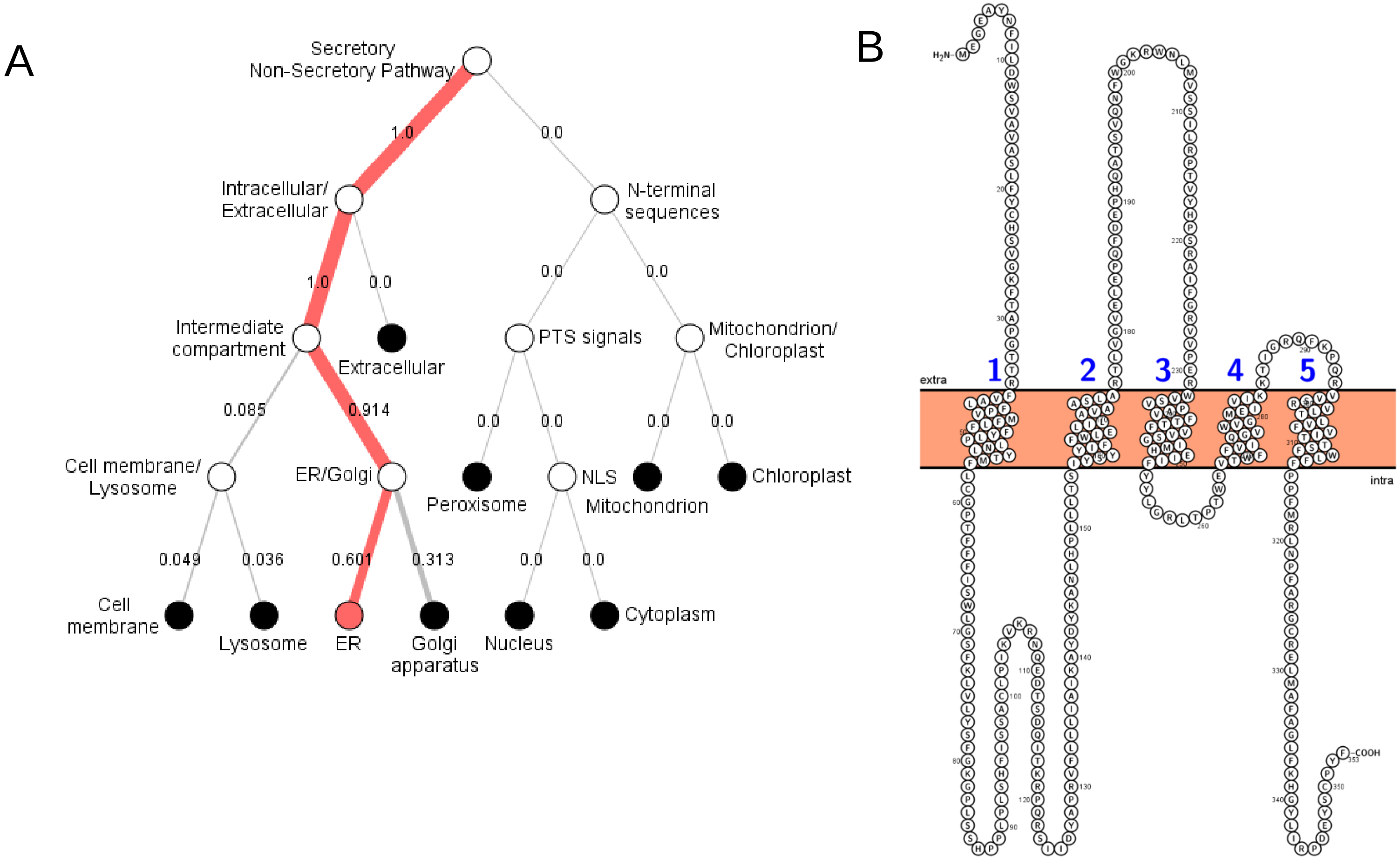

Figure S2. Estimation of cellular localization and membrane topology of the LsTAT1 enzyme. The subcellular localization of the LsTAT1 enzyme (A) was analysed using DeepLoc-1.0 (Almagro Armenteros et al. 2017). A proposed model (B) for LsTAT1 topology on the ER membrane was constructed using Protter software (Omasits et al., 2014).
