# Supplementary material for: Characterization of a Pentacyclic Triterpene Acetyltransferase Involved in the Biosynthesis of Taraxasterol and ψ-Taraxasterol Acetates in Lettuce
Source: Front Plant Sci. 2022 Jan 3;12:788356. doi: 10.3389/fpls.2021.788356 (PMC8762322; doi:10.3389/fpls.2021.788356)
Supplement: Supplementary file 3 [file Data_Sheet_3.PDF]

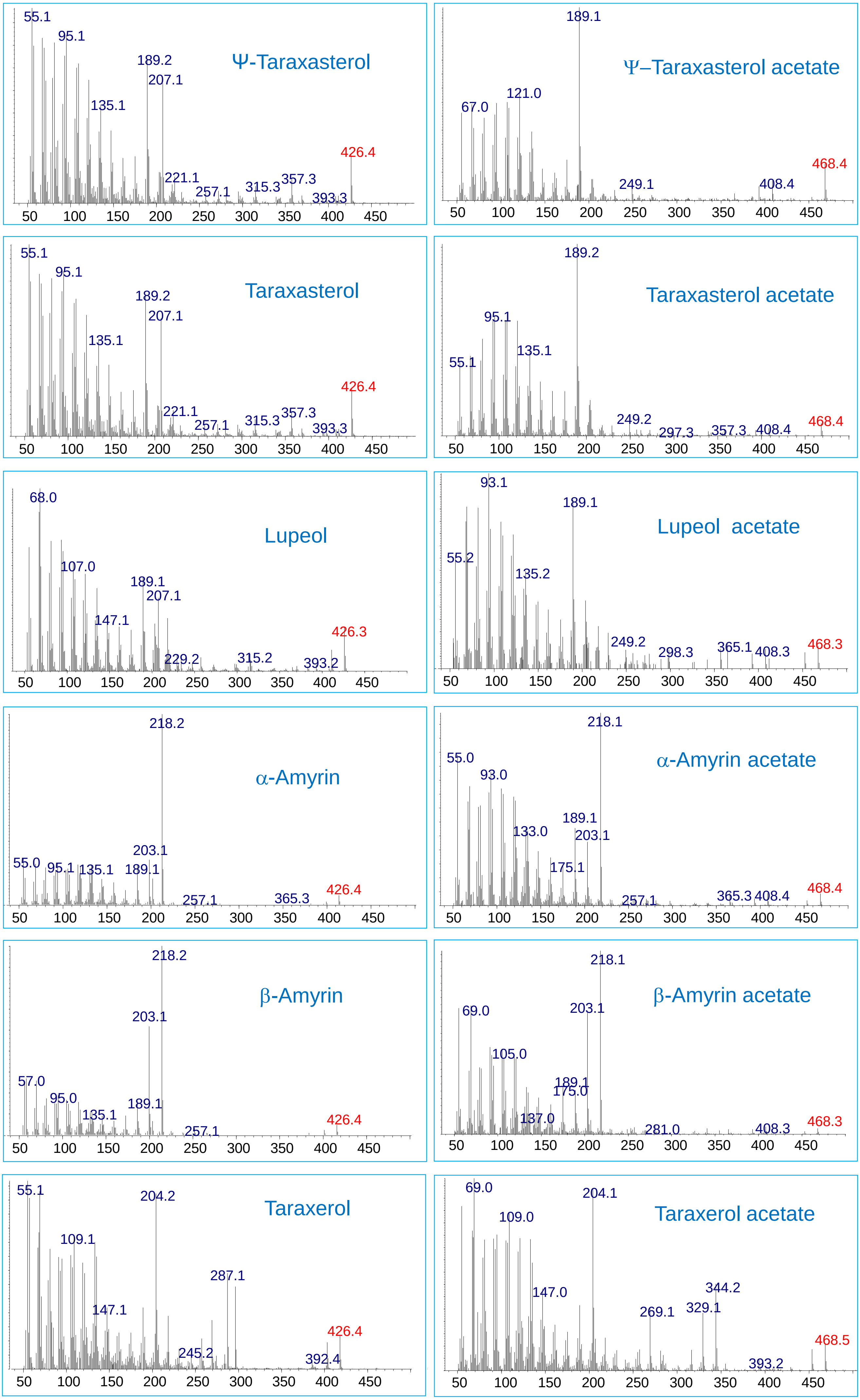

Figure S3. MS spectra for the triterpene and triterpene acetate peaks detected in the GC chromatogram of the LsTAT1 enzyme reaction with various triterpenes.
