# Supplementary material for: Characterization of a Pentacyclic Triterpene Acetyltransferase Involved in the Biosynthesis of Taraxasterol and ψ-Taraxasterol Acetates in Lettuce
Source: Front Plant Sci. 2022 Jan 3;12:788356. doi: 10.3389/fpls.2021.788356 (PMC8762322; doi:10.3389/fpls.2021.788356)
Supplement: Supplementary file 4 [file Data_Sheet_4.PDF]

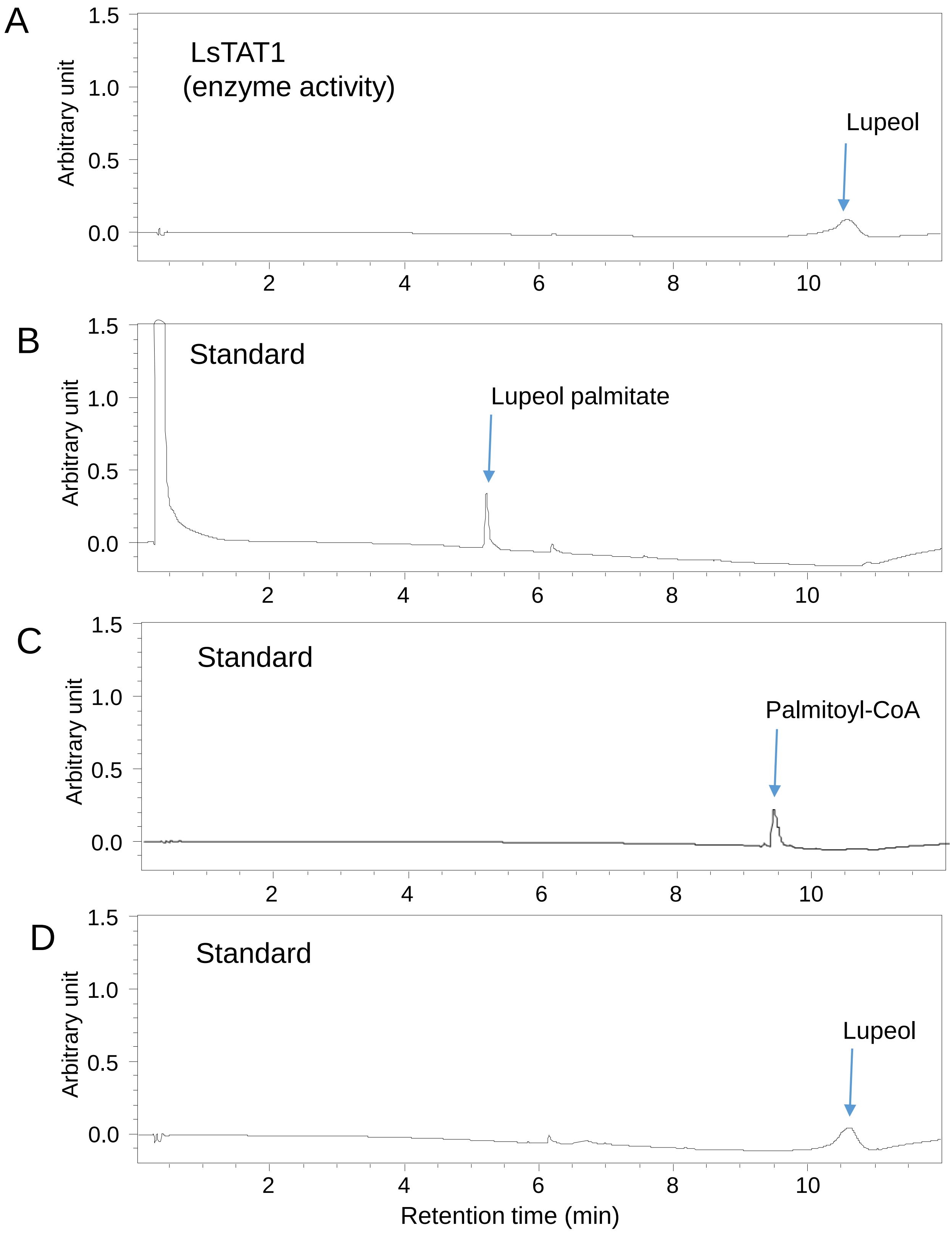


Figure S4. In vitro activity of the LsTAT1 enzyme on the conversion of lupeol to lupeol palmitate by the reaction of palmitoyl-CoA (16:0) as an acyl donors and lupeol as an acyl accepter. (A) UPLC chromatograms of the reaction products of the LsTAT1 enzyme with lupeol and palmitoyl-CoA. (B-D) UPLC chromatograms of standards (lupeol, palmitoyl-CoA, and lupeol palmitate).
