# Supplementary material for: Characterization of a Pentacyclic Triterpene Acetyltransferase Involved in the Biosynthesis of Taraxasterol and ψ-Taraxasterol Acetates in Lettuce
Source: Front Plant Sci. 2022 Jan 3;12:788356. doi: 10.3389/fpls.2021.788356 (PMC8762322; doi:10.3389/fpls.2021.788356)
Supplement: Supplementary file 5 [file Data_Sheet_5.PDF]

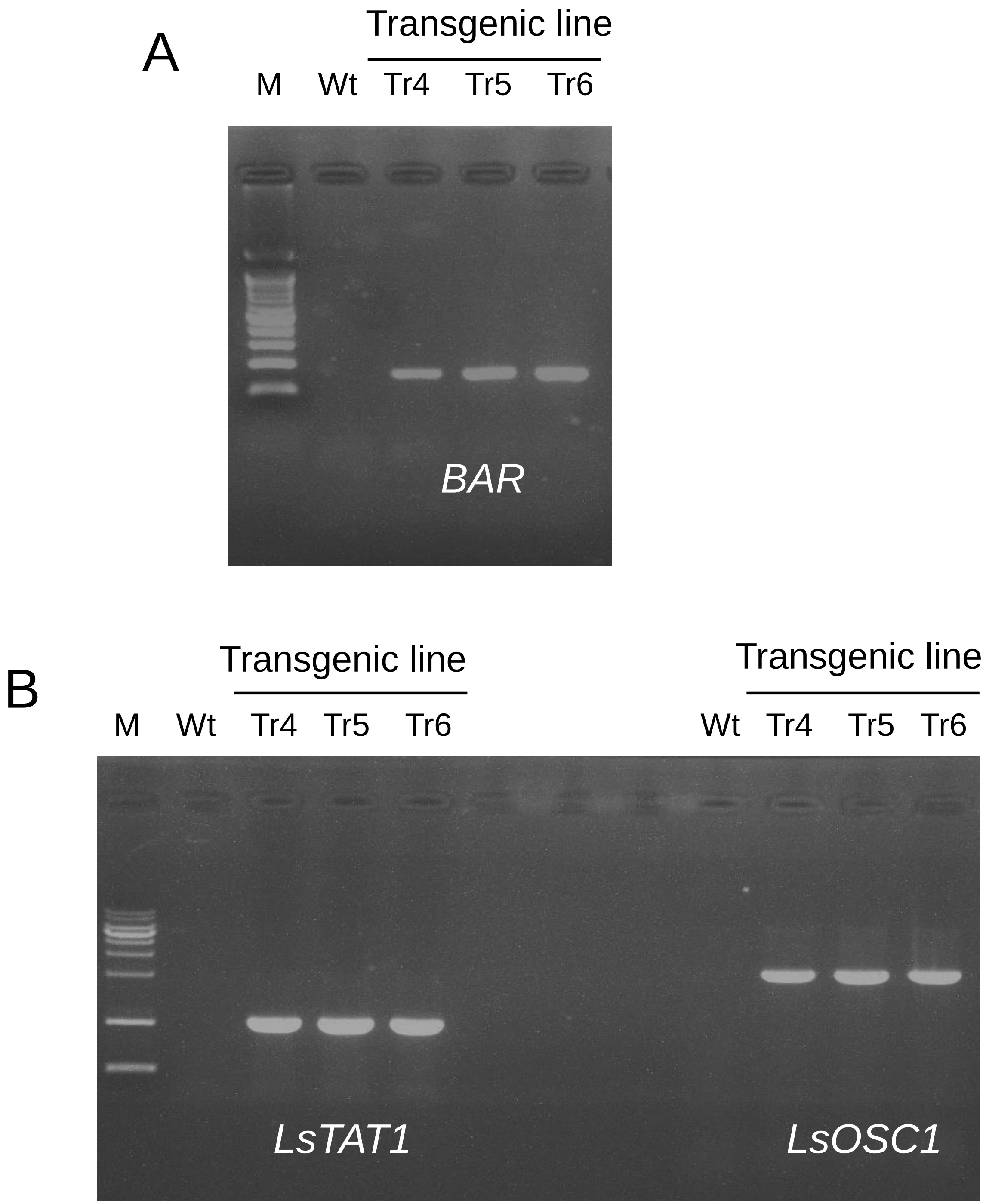

Figure S5. Genomic PCR amplification of introduced selection marker (*BAR*) (A), and *LsOSC1* and *LsTAT1* genes (B) in wild-type and transgenic tobacco lines.
