# Supplementary material for: Characterization of a Pentacyclic Triterpene Acetyltransferase Involved in the Biosynthesis of Taraxasterol and ψ-Taraxasterol Acetates in Lettuce
Source: Front Plant Sci. 2022 Jan 3;12:788356. doi: 10.3389/fpls.2021.788356 (PMC8762322; doi:10.3389/fpls.2021.788356)
Supplement: Supplementary file 6 [file Data_Sheet_6.PDF]

Supplementary Data

Table S1: Primers used for amplification of genes in the study.

Primers	Direction	Sequences
LsOSC1 for yeast and tobacco expression	Forward	5'-ATG TGG AAG CTC AAA ATA G-3'
	Reverse	5'-GGA GTT TAG TTT TCT TGT TT-3'
LsTAT1 for yeast and tobacco expression	Forward	5'- CGT GCT TAT AGA ATT ATC GAT TT-3'
	Reverse	5'- ATA AAT GTG TGT TCT CTT TTC AT-3'
XM023877741.	Forward	5`- CGG TCC TAC CTC TTT CTT CGT A-3`
	Reverse	5`- TCG TCG GAT TTT CCA TCT TGA G-3`
XM023886997.1	Forward	5`- TCA AAC TGC TGT TGT ATG CGT-3`
	Reverse	5`- AGA TTT TCC TTC TGA TCG GCG-3`
XM023884745.1	Forward	5`- TTC AAC ATC GGT GAT GGA AAT GG-3`
	Reverse	5`- TTG TTC TCG AAA CCT TTC AAC TTG T-3`
XM023879520.1	Forward	5`- CCG CTG TTT TCA CGA CGT TT-3`
	Reverse	5`- GGG GCT TAA ACT GAC GAC CA-3`
XM023879494.1	Forward	5`- CCG CTG TTT TCA CGA CGT TT-3`
	Reverse	5`- GGG GCT TAA ACT GAC GAC CA-3`
XM023872407.1	Forward	5`- TTT GGC TTT CGT GTT GGC AA-3`
	Reverse	5`- TAC TGG CGT AGT TCG TAC CAC-3`
XM023887114.1	Forward	5`- AAA TAG GAC CCC TTT CCG CC-3`
	Reverse	5`- CCA AGC GAT CAT GGG GTG TA-3`
XM023885793.1	Forward	5`- TTG AGC TCG TTC TCG CGA T-3`
	Reverse	5`- TAC ACA GTA GGC CGG AGG AT-3`
XM023888915.1	Forward	5`- CAA GGC CCA CTT TAT CCC TCT-3`
	Reverse	5`- ACT AGT GCA CAG TAG ATG CCT-3`
XM023885785.1	Forward	5`- TCC GAT CAT AAA TGG GCC CC-3`
	Reverse	5`- ACC AGC CAG ATT GCT CTT GA-3`
β-Actin	Forward	5'- CGG AAT TGT CAG CAA CTG GG -3′
	Reverse	5'- CTT CAG TGA GAA GAA CAG GGT GT-3′
